# Induction of seizures and initiation of epileptogenesis by pilocarpine in zebrafish larvae

**DOI:** 10.3389/fnmol.2024.1418606

**Published:** 2024-08-06

**Authors:** Kinga Gawel, Monika Hulas-Stasiak, Marta Marszalek-Grabska, Anna Grenda, Aleksandra Siekierska, Nataliia Kosheva, Wietske van der Ent, Camila V. Esguerra, Pawel Krawczyk, Waldemar A. Turski

**Affiliations:** ^1^Department of Experimental and Clinical Pharmacology, Medical University of Lublin, Lublin, Poland; ^2^Department of Functional Anatomy and Cytobiology, Maria Curie-Sklodowska University, Lublin, Poland; ^3^Department of Pneumology, Oncology and Allergology, Medical University of Lublin, Lublin, Poland; ^4^VirusBank Platform, Department of Microbiology, Immunology and Transplantation, KU Leuven, Leuven, Belgium; ^5^Chemical Neuroscience Group, Centre for Molecular Medicine Norway, University of Oslo, Forskningsparken, Oslo, Norway

**Keywords:** zebrafish, pilocarpine, seizure, epileptogenesis, locomotor activity, local field potential recordings

## Abstract

**Objective:**

Preclinical models of seizures and epilepsy in rodents contributed substantially to the discovery of currently available antiseizure medications. These were also broadly used for investigation of processes of epileptogenesis. Nevertheless, rodent models pose some limitations, thus, new models using alternative species are in high demand. The aim of this study was to describe a new model of seizures/epilepsy induced by the cholinomimetic agent, pilocarpine (PILO), in larval zebrafish.

**Methods:**

Local field potential (LFP) recordings were conducted to analyze electroencephalographic discharges and correlate it with larval behavior. Hematoxylin and eosin (H&E) staining, as well as TUNEL staining were performed to analyze morphology and apoptosis, respectively. Real-time quantitative polymerase chain reaction (qRT-PCR) was undertaken for gene expression analysis.

**Results:**

Acute exposure to PILO, in a concentration-dependent manner, induces electroencephalographic discharges in larval zebrafish, which behaviorally manifest as decreased locomotion and moving time, but enhanced movement velocity. The PILO-induced seizure-like activity is behaviorally distinct from this induced by the application of chemoconvulsant pentylenetetrazole (PTZ). Zebrafish larvae previously exposed to PILO (2 h), after a washing out period, exhibit spontaneous, unprovoked discharges and apoptotic changes in their brains.

**Significance:**

Here, we comprehensively investigated a new model of PILO-induced seizures/epilepsy in larval zebrafish. We propose that this model may be used to study epileptogenesis and for antiseizure drug screening purposes.

## 1 Introduction

In the early 1980s, a new animal model of convulsions induced by pilocarpine (PILO) was established (Turski et al., 1983a, 1983b, 1984). It was then shown that PILO and other cholinomimetics administered intracerebrally or peripherally induce repetitive seizures that progress to *status epilepticus* in rodents. Moreover, the investigations demonstrated that electroencephalographic alterations are accompanied by characteristic behavioral changes that resemble temporal lobe epilepsy in humans. Unlike other models of chemically-induced convulsions, recurrent convulsions and *status epilepticus* induced by PILO result in morphological changes in the brain, primarily in the limbic system (Turski et al., 1983a, 1984, 1989). The next milestone was the demonstration of the presence of unprovoked paroxysmal electroencephalographic changes accompanied by behavioral seizures, which occur in animals long time after the end of the acute phase of convulsions (Cavalheiro et al., 1991, 1994). It was found that the spontaneous recurrent seizures are preceded by a seizure-free period lasting from 1–7 weeks, and then persist throughout the life of the animal (Cavalheiro et al., 1991, 1994). This silent period is considered to be the time when the process of epileptogenesis occurs.

The uniqueness of PILO-induced convulsions in rodents lies in putting into the hands of researchers an animal model that allowed the exploration of the molecular mechanisms of epileptogenesis and initiated experimental studies aimed at searching for drugs that modify this process.

Nowadays, studies performed on zebrafish, especially on their larvae, are steadily growing in popularity (Baraban, 2007). The data collected demonstrated that either gene manipulation or exposure of larvae to chemoconvulsants results in convulsions (Stewart et al., 2012). It is also possible to screen for potential anticonvulsants (Knap et al., 2023). For this purpose, pentylenetetrazole (PTZ) is most often used in zebrafish research. Although convulsions induced by cholinomimetics were reported in several publications (Park et al., 2008; Vermoesen et al., 2011; Koenig et al., 2016; Lopes et al., 2016; Winter et al., 2017; Paudel et al., 2020; Szep et al., 2023), PILO-induced convulsions have not been thoroughly analyzed and remain poorly understood. Therefore, our aim was to examine whether PILO exposure to zebrafish larvae causes paroxysmal electroencepholographic and behavioral changes, and whether it results in the induction of epileptogenesis, morphological changes in the brain and the appearance of unprovoked seizures.

## 2 Materials and methods

### 2.1 Chemicals

PILO, PTZ and diazepam (DZP) were purchased from Sigma-Aldrich (St. Louis, MO, United States). PILO stock solution (150 mM) was prepared each time *ex tempore*. The following final concentrations of PILO were applied 1, 10, 30, 50 or 100 mM. PTZ was dissolved in MilliQ water to create a 60 mM stock. DZP (stock solution 10 mM) was dissolved in ethanol and employed in final concentration of 10 or 20 μM.

### 2.2 Zebrafish husbandry

At the Centre for Molecular Medicine Norway (NCMM), University of Oslo, Norway, zebrafish larvae of AB background were the subject of local field potential (LFP) recordings. In the other studies, the Centre for Experimental Medicine, Medical University of Lublin, Poland provided zebrafish embryos of the same strain as above. In the two laboratories, breeding of embryos and larvae occurred in E3 medium: 1.5 mM HEPES (pH 7.6), 17.4 mM NaCl, 0.21 mM KCl, 0.12 mM MgSO_4_, 0.18 mM Ca(NO_3_)_2_, with 6 μM methylene blue (Sigma-Aldrich) as antifungal agent. Larvae were kept in dedicated incubators (temperature 28.5 ± 1°C, 14 h light: 10 h dark photoperiod). Immediately after experiments, larvae were euthanized via 15 μM of tricaine (Sigma-Aldrich).

In the experiments, the larvae age did not exceed 120 h post-fertilization (hpf). Hence, as per current European Union and Norwegian legislation, ethical permission for experimental purposes was not required. The experimenters made all possible efforts to avoid suffering so as to be in compliance with National Institute of Health Guidelines for the Care and Use of Laboratory Animals and the European Community Council Directive of November 2010 for Care and Use of Laboratory Animals (Directive 2010/63/EU). ARRIVE guidelines were followed for animal reporting. Observers were blind to treatment groups.

### 2.3 Toxicity assessment

Exposure to PILO occurred at concentrations of 10, 30, 50, and 100 mM for 2 h (*N* = 32/group). Viable larvae count was undertaken 2 h and 22 h post-exposure. The larvae were washed-out 5 times in fresh medium (without PILO) and maintained in regular medium (without PILO). All assessments were carried out under light (by a trained observer).

### 2.4 Local field potential recordings

Each larva was mounted on a glass slide, which was subsequently covered with a thin layer of 2% agarose, low gelling temperature (Sigma-Aldrich). A glass electrode (resistance 1–5 MΩ) filled with artificial cerebrospinal fluid (124 mM NaCl, 2 mM KCl, 2 mM MgSO_4_, 2 mM CaCl_2_, 1.25 mM KH_2_PO_4_, 26 mM NaHCO_3_, 10 mM glucose) was placed into the *optic tectum* (a part of the midbrain), and signals recorded by way of a Digidata 1,550 with a MultiClamp 700B amplifier (Axon Instruments, United States). Each recording lasted for at most 20 min. Data processing occurred via Clampfit 10.2 software (Molecular Devices Corporation, United States).

### 2.5 Locomotor activity

Locomotor activity measurement and data processing utilized Noldus tracker device (Wageningen, Netherlands) and the EthoVision XT programme, respectively. Individual larvae were placed in wells with medium (48-well plates) and accommodated to apparatus for 15 min. Afterwards, PILO, PTZ or medium was added to reach final concentrations. After 5 min, tracking was undertaken for 60 or 120 min (1 min time intervals), and distance traveled and moving time were recorded (mm/s). Movement velocity was then calculated (distance traveled divided by moving time). In the light–dark transition assay, distance traveled was recorded for four subsequent phases of 10 min, each: 100% light–dark—100% light–dark.

### 2.6 Histology

Larvae were cooled down and fixed in 10% buffered formalin for 24 h at room temperature. Subsequently, they were dehydrated and embedded in Paraplast (Sigma-Aldrich, St. Louis, MO, United States). Samples were serially sectioned (5 μm thickness), then mounted on polysine-coated glass slides (Thermo Fisher Scientific, Braunschweig, Germany) according to standard procedure. Hematoxylin and eosin (H&E) stained slides were examined by means of an Axiovert 200 M confocal microscope coupled with a digital camera AxioCamHRc and software Axio Vision 4.8 (Carl Zeiss, Jena, Germany).

TUNEL assay was performed according to manufacturer’s protocol employing an ApopTag^®^ Peroxidase *In Situ* Apoptosis Detection Kit (Chemicon International, Melbourne, Australia). Negative control consisted of sections fixed without active enzyme. Cells stained dark brown were interpreted as TUNEL-positive apoptotic cells. All slides were examined and photographed using an Axiovert 200 M confocal microscope coupled with a digital camera AxioCamHRc and software Axio Vision 4.8 (Carl Zeiss, Jena, Germany). Midbrain apoptotic cell count was undertaken by way of public domain ImageJ (Wayne Rasband, National Institute of Mental Health, Bethesda, MD, United States) (3 sections per larva, *N* = 7 larvae/group). All results were expressed as percentage of apoptotic cells over total cell number per section per larva.

### 2.7 Expression of genes on mRNA level

Exposure to PILO (50 mM) took place for 2 h. The larvae were then thoroughly washed out five times and, afterwards, incubated in standard medium for 22 h. They were then subjected to analysis (*N* = 6 samples/group, *N* = 25 larvae/sample). Isolation of RNA was undertaken using PureLink^™^ RNA Mini Kit (Thermo Fisher Scientific, Waltham, Massachusetts, United States) via manufacturer’s instruction. Performance of reverse transcription reactions was by way of a High-Capacity cDNA Reverse Transcription Kit (Thermo Fisher Scientific, Waltham, Massachusetts, United States) per manufacturer’s instruction. Quantitative PCR reaction was determined utilizing GoTaq^®^ Master Mix (Promega, Madison, Wisconsin, United States). In the work, *13 ribosomal (18S rRNA)* and *beta-2-microglobulin* acted as internal control (housekeeping genes). Primer sequences were:

**Table 1:** 

Gene name	Forward primer	Reverse primer	Source
*casp3a*	ATGAACGGAGACTGTGTG	TTAAGGAGTGAAGTACATCTCTTTG	Spead et al. (2018)
*casp3b*	ATGTCGCACGTGAAACCA	TTATTTAGGGAAGTAGAGTTCTTTGG	
*npas4a*	AGCCAAGTCTGCCCTTCTTCT	TGCTGTGCTAAAAGCGAGATCT	Klaric et al. (2014)
*18S rRNA*	TCGCTAGTTGGCATCGTTTATG	CGGAGGTTCGAAGACGATCA	McCurley and Callard (2008)
*β2-microglobulin*	GCCTTCACCCCAGAGAAAGG	GCGGTTGGGATTTACATGTTG	

All qPCR reactions were duplicated. Relative expression was determined by applying 2^−∆∆Ct^ method.

### 2.8 Statistical analysis

Experimental data in this study are manifested as median, mean ± standard deviation (SD) or percentage as indicated in the legends, and statistical comparisons were performed. Statistical significance level was set at *p* < 0.05. Statistica (version 13.3) software or GraphPad Prism 9.3.1 version (San Diego, CA, United States) were employed in the evaluations.

## 3 Results

### 3.1 Pilocarpine-induced toxicity

A 2 h exposure to PILO (100 mM) resulted in the death of 81.25% of the larvae; for ethical reasons, all remaining larvae in this group were euthanized. Exposure to PILO up to concentration of 50 mM did not cause the death of any larvae after 2 h and 24 h. Therefore, in further studies, PILO was used up to 50 mM.

### 3.2 Acute exposure to pilocarpine induces seizure-like LFP events

**Figure 1:**
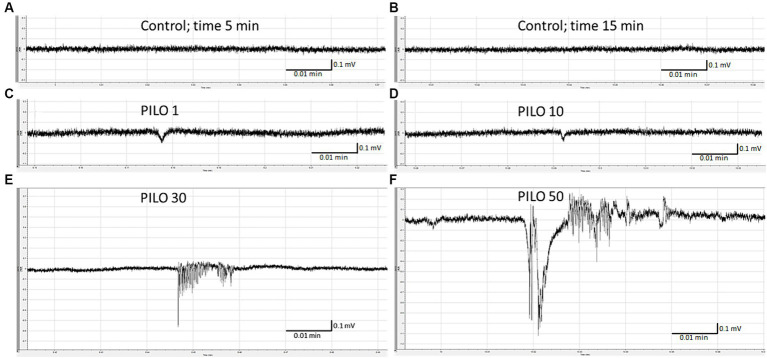
Local field potentials (LFPs) recorded from the brain of zebrafish larvae exposed to pilocarpine (PILO) at different concentrations. Larvae were incubated in different concentrations of PILO (1, 10, 30 or 50 mM) for 5 min, and, afterwards, LFPs were recorded for 20 min: **(A,B)** control recording; **(C–F)** representative pattern of electrographic events recorded after acute exposure of larvae to PILO.

**Figure 2:**
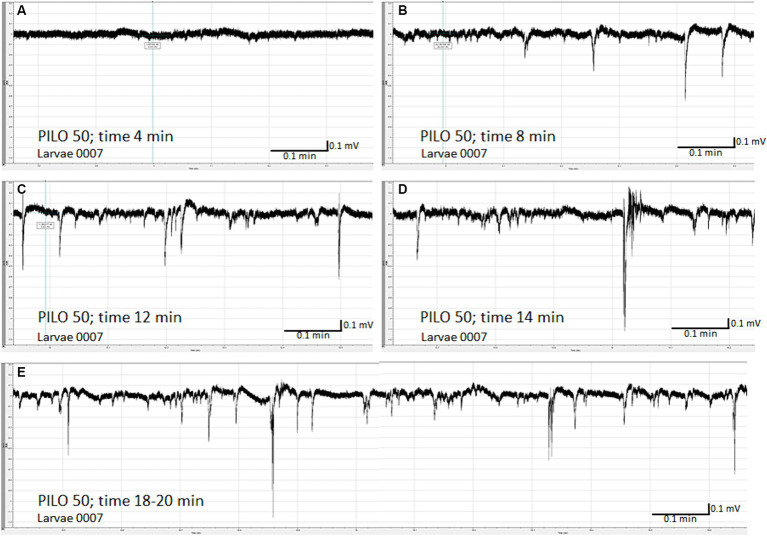
Time-dependent evolution of electroencephalographic changes recorded from the brain of zebrafish larvae exposed to pilocarpine (PILO). Larvae was incubated in medium containing PILO (50 mM) for 5 min, and, afterwards, LFPs were recorded for 20 min: **(A)** 4 min after start of recording, no clear-cut seizure-like events are present; **(B)** 8 min after start of recording, short ictal and interictal events are seen; **(C)** 12 min after start of recording; progression of seizure-like ictal and interictal activity is evidenced **(D)** 14 min after start of recording, further progression and intensification of seizure-like ictal and interictal activity is shown; **(E)** 18–20 min after start of recording, prolonged and continuous convulsion-like activity.

LFP recording of brain activity revealed that acute exposition of larvae to PILO results in seizure-like activity. The effect was concentration-dependent. No seizure-like activity was recorded in control (Figures 1A,B) and in larvae exposed to PILO at concentrations of 1 and 10 mM (Figures 1C,D). Seizure-like activity was found in larvae exposed to 30 mM PILO (Figure 1E). Ictal events recurred repeatedly several times during the 20 min recording. In larvae exposed to 50 mM PILO, ictal activity increased over time, seizures had greater amplitude and lasted longer (Figure 1F). Finally, continuous convulsion-like activity occurred in most of the larvae tested (Figures 2A–E).

### 3.3 Diazepam counteracts pilocarpine-induced seizure-like LFP events

**Figure 3:**
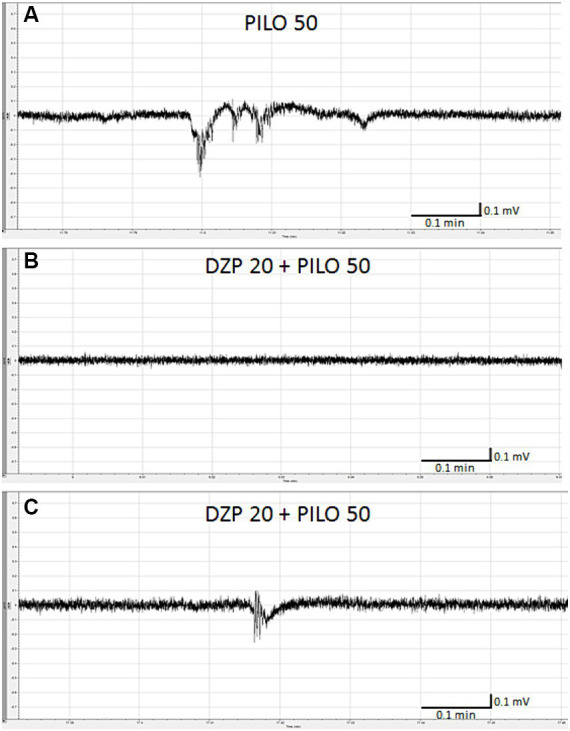
Effect of diazepam (DZP) on PILO-induced local field potential (LFP) events. Larvae were incubated in medium containing DZP (20 μM) for 20 h, and, subsequently, PILO was added to wells (final concentration = 50 mM). After 5 min incubation with PILO, LFPs recording were performed: **(A)** control recording of seizure-like events as presented on the graph evoked by PILO 50 mM; **(B)** representative LFP recordings performed in larvae exposed to DZP 20 μM + PILO 50 mM, note that in 3 out of 4 larvae, no seizure-like activity was recorded within 20 min of analysis, **(C)** only 2 short-lasting, low amplitude seizure-like events were found in larvae no. 3 within 20 min of analysis.

Pre-exposure of zebrafish larvae to DZP in concentration of 20 μM for 22 h prevented and/or inhibited seizure-like electrographic events induced by 50 mM PILO in 3 out of 4 larvae (Figures 3A,B). Only two, short-lasting low-amplitude episodes of ictal activity was recorded in 1 out of 4 larvae (Figure 3C). DZP in concentration of 10 μM did not prevent seizure-like electrographic events induced by 50 mM PILO in 5 out of 5 larvae (data not shown).

### 3.4 Spontaneous seizure-like activity caused by prior exposure of zebrafish larvae to pilocarpine

**Figure 4:**
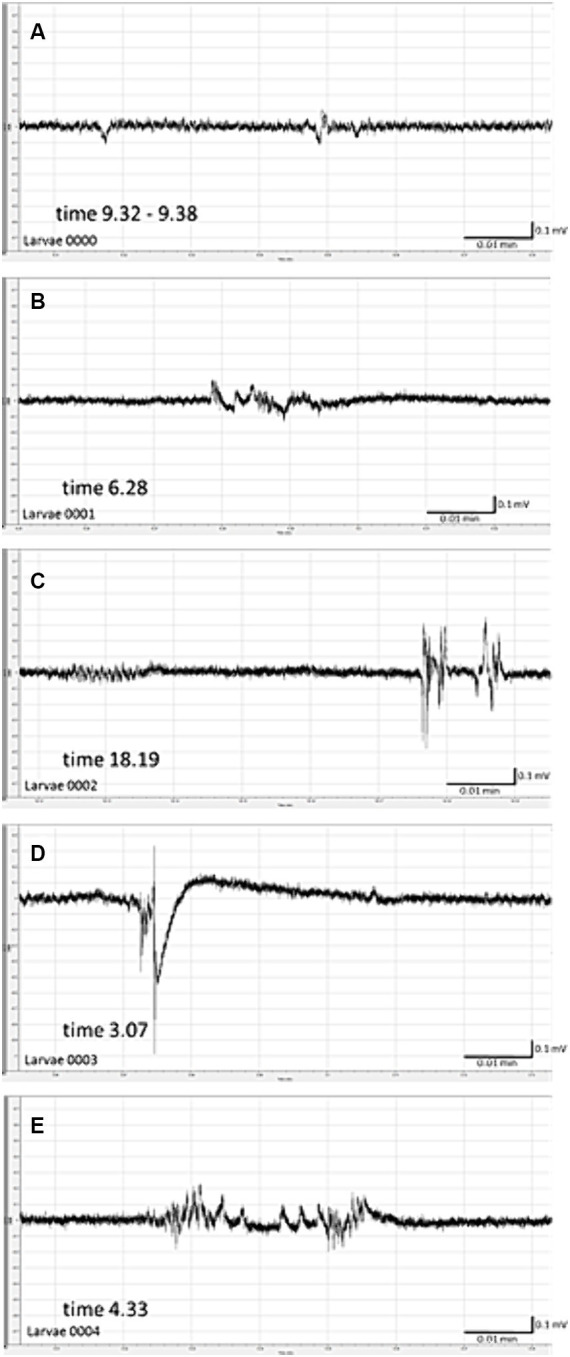
Spontaneous local field potentials (LFPs) recorded from the brain of zebrafish larvae previously exposed to pilocarpine (PILO). Larvae were incubated in medium containing PILO (50 mM) for 120 min, and, afterwards, larvae were thoroughly washed out 5 times in fresh medium and incubated in PILO-free medium for the next 22 h. Subsequently, LFP recordings were performed: **(A–E)** representative pattern of electrographic events recorded 22 h after acute exposure to PILO (50 mM). Note unprovoked, recurrent abrupt seizure-like events with miscellaneous patterns, duration and amplitude.

Spontaneous seizure-like activity was recorded 22 h after cessation of exposure of larvae to PILO 50 mM for 2 h (Figures 4A–E). All 5 larvae tested experienced unprovoked, repetitive paroxysmal alternations of brain activity resembling isolated seizures (Figures 4A–E). The incidence of seizure-like LFP events ranged from 3 to 12 per 20 min of recording per larvae. There was no continuous convulsive activity observed in any larva.

### 3.5 Acute exposure to pilocarpine reduces mobility of zebrafish larvae

**Figure 5:**
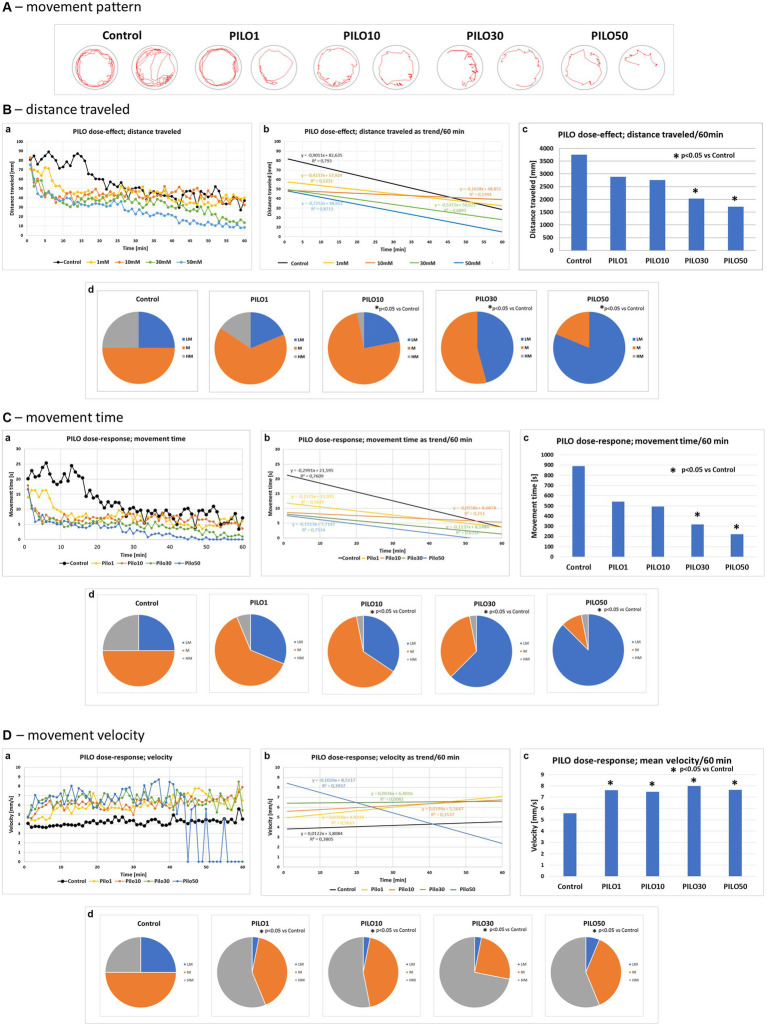
Locomotor activity of zebrafish larvae acutely exposed to pilocarpine (PILO). Larvae were incubated in different concentrations of PILO (1, 10, 30 or 50 mM) for 5 min and afterward locomotor activity assay was conducted for 60 min: **(A)** representative track visualizations which show the manner and intensity of zebrafish larvae movement in the well area (red line); the analysis was conducted at 9–10 min interval. **(B,a)** Each point represents median value of distance traveled at 1 min intervals; **(b)** locomotion was presented as a linear trend—the equation *y* = *mx* + c and squared value *R*^2^ are given in the graph; **(c)** total distance traveled in 60 min (data are presented as median values)—statistical analysis was performed using nonparametric ANOVA Kruskal–Wallis test, followed by Dunn’s Multiple Comparisons test (significance level was set at *p* < 0.05); **(d)** the pie charts show the fractions of larvae in quartiles: LM—larvae with low total mobility, M—larvae with medium total mobility, HM—larvae with high total mobility—statistical analysis was performed using chi-square (*χ*^2^) statistics (significance level was set at *p* < 0.05, number of subjects analyzed *N* = 32/group). **(C,a)** Each point represents median value of movement time registered at 1 min intervals; **(b)** movement time was presented as a linear trend; **(c)** total distance traveled in 60 min; **(d)** the pie charts show the fractions of larvae in quartiles; presentation of results and statistical analysis as described in **(B,a)**. **(D,a)** Each point represents median value of movement velocity registered at 1 min intervals; **(b)** movement velocity presented as a linear trend; **(c)** mean movement velocity traveled in 60 min; **(d)** the pie charts show the fractions of larvae in quartiles: LM—larvae with low total mobility, M—larvae with moderate total mobility, HM—larvae with high total mobility; presentation of results and statistical analysis as described in **(B,a)**.

Acute exposure of zebrafish larvae to PILO caused a concentration-dependent alternation of movement pattern (Figure 5A). A decrease in larval motility measured as distance traveled (Figure 5B,a–c) and movement time was revealed (Figure 5C,a–c). In contrast, movement velocity of larvae exposed to all tested concentrations of PILO was significantly enhanced (Figure 5D,a–c). It was evident that after 40 min of exposure to PILO 50 mM, a large proportion of the larvae remained immobile during the 1 min recording intervals (Figure 5D,a). When the movement pattern of the larvae was observed under the microscope, it was found that larvae exposed to 50 mM PILO exhibited paroxysmal, fast, short-lasting, short-distance movements and that such movements occurred repeatedly. The quartile analysis revealed that acute exposition to PILO concentration-dependently reduced the proportion of subjects traveling longer distance and spending more time moving. Significant changes were found in groups exposed to 30 and 50 mM PILO (Figures 5B,C, d). An inverse trend occurred in the number of fast-moving larvae, which increased as the concentration of PILO increases from 1 to 10 mM (Figure 5D,d).

### 3.6 Behavioral consequences registered 22 h after prior exposure of zebrafish larvae to pilocarpine

**Figure 6:**
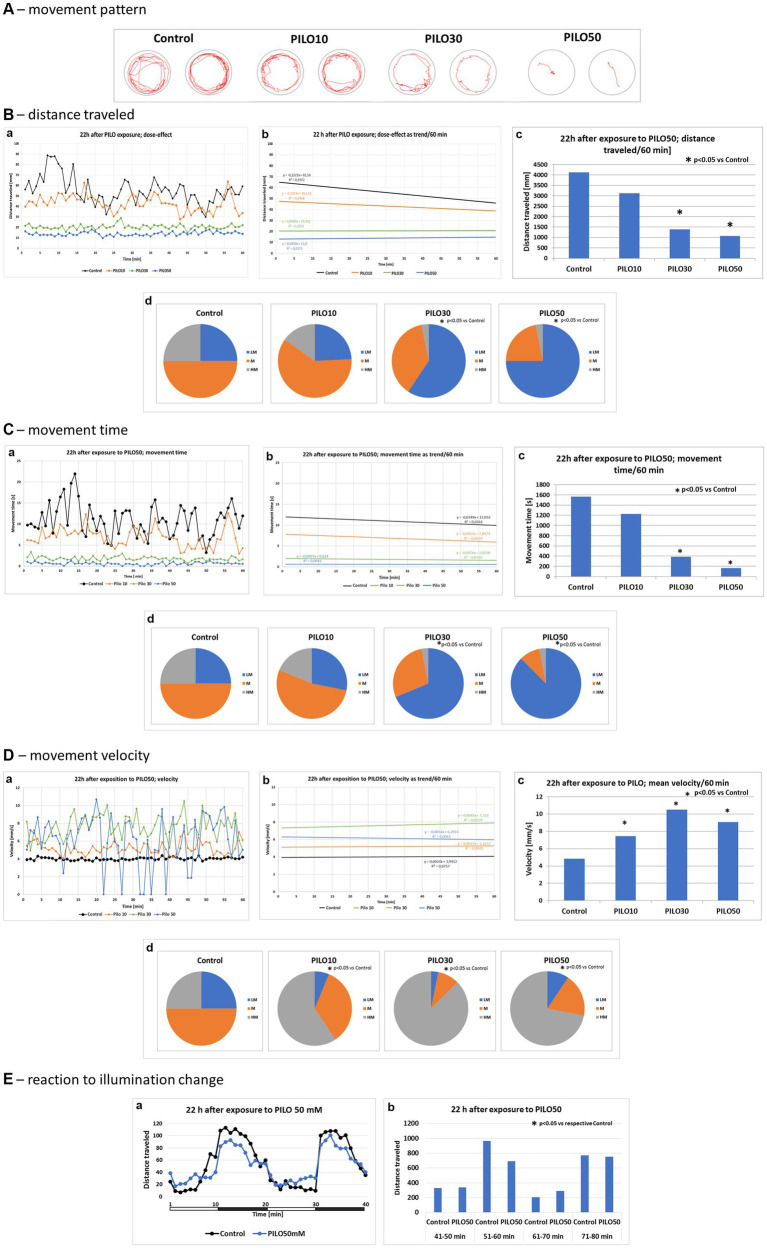
Locomotor activity of zebrafish larvae 22 h after exposition to pilocarpine (PILO). Larvae were incubated in different concentrations of PILO (10, 30 or 50 mM) for 120 min. Next, larvae were thoroughly washed out 5 times, and incubated in fresh medium for subsequent 22 h. Afterwards, locomotor activity assay was conducted for 120 min: **(A)** representative track visualizations which show the manner and intensity of zebrafish larvae movement in the well area (red line)—the analysis was conducted at 9–10 min interval. **(B,a)** Each point represents median value of distance traveled at 1 min intervals; **(b)** locomotion was presented as a linear trend; the equation *y* = *mx* + c and squared value *R*^2^ are given in the graph; **(c)** total distance traveled in 60 min (data are presented as median values)—statistical analysis was performed using nonparametric ANOVA Kruskal–Wallis test, followed by Dunn’s Multiple Comparisons test (significance level was set at *p* < 0.05); **(d)** the pie charts show the fractions of larvae in quartiles: LM—larvae with low total mobility, M—larvae with medium total mobility, HM—larvae with high total mobility; statistical analysis was performed using chi-square (*χ*^2^) statistics (significance level was set at *p* < 0.05, number of subjects analyzed was *N* = 32 in control groups and in each PILO exposed group). **(C,a)** Each point represents median value of movement time registered at 1 min intervals; **(b)** movement time was presented as a linear trend; **(c)** total distance traveled in 60 min; **(d)** the pie charts show the fractions of larvae in quartiles; presentation of results and statistical analysis as described in **(B,a)**. **(D,a)** Each point represents median value of movement velocity registered at 1 min intervals; **(b)** movement velocity presented as a linear trend; **(c)** mean movement velocity traveled in 60 min; **(d)** the pie charts show the fractions of larvae in quartiles: LM—larvae with low total mobility, M—larvae with moderate total mobility, HM—larvae with high total mobility; presentation of results and statistical analysis as described in **(B,a)**. **(E)** Dark–light assay was conducted with following phases: 100% light–100% dark—100% light–100% dark, 10 min each: **(a)** each point represents median value of distance traveled at 1 min intervals; horizontal white-black bar illustrates the sequence of illumination changes, light and darkness alternately; **(b)** total distance traveled in 10 min intervals; horizontal white-black bar illustrates the sequence of illumination changes, light and darkness alternately (data are presented as median values)—statistical analysis was performed using nonparametric ANOVA Kruskal–Wallis test, followed by Dunn’s Multiple Comparisons test (significance level was set at *p* < 0.05, number of subjects analyzed was *N* = 16/control group and *N* = 32/each PILO exposed group).

Exposure of larvae to PILO (10–50 mM) for 2 h resulted in a concentration-dependent alternation of their movement pattern as recorded 22 h later (Figure 6A). PILO 10 mM did not significantly affect distance traveled and movement time of larvae (Figures 6B,C, a–d). At both 30 and 50 mM, PILO reduced distance traveled and movement time at 22 h post-exposure (Figures 6B,C, a–d). Movement velocity of larvae exposed to 10–50 mM PILO was significantly enhanced. Noticeable is the very high fluctuation in the speed of movement of larvae (Figure 6D,a–d). Prior exposure to PILO (50 mM) did not significantly affect the response of larvae to a change in illumination (Figure 6E,a,b), which confirms the ability of larvae to respond to stimuli and points to unimpaired ability to perform movement.

### 3.7 Comparison of locomotor activity of zebrafish larvae exposed to pilocarpine or pentylenetetrazole

**Figure 7:**
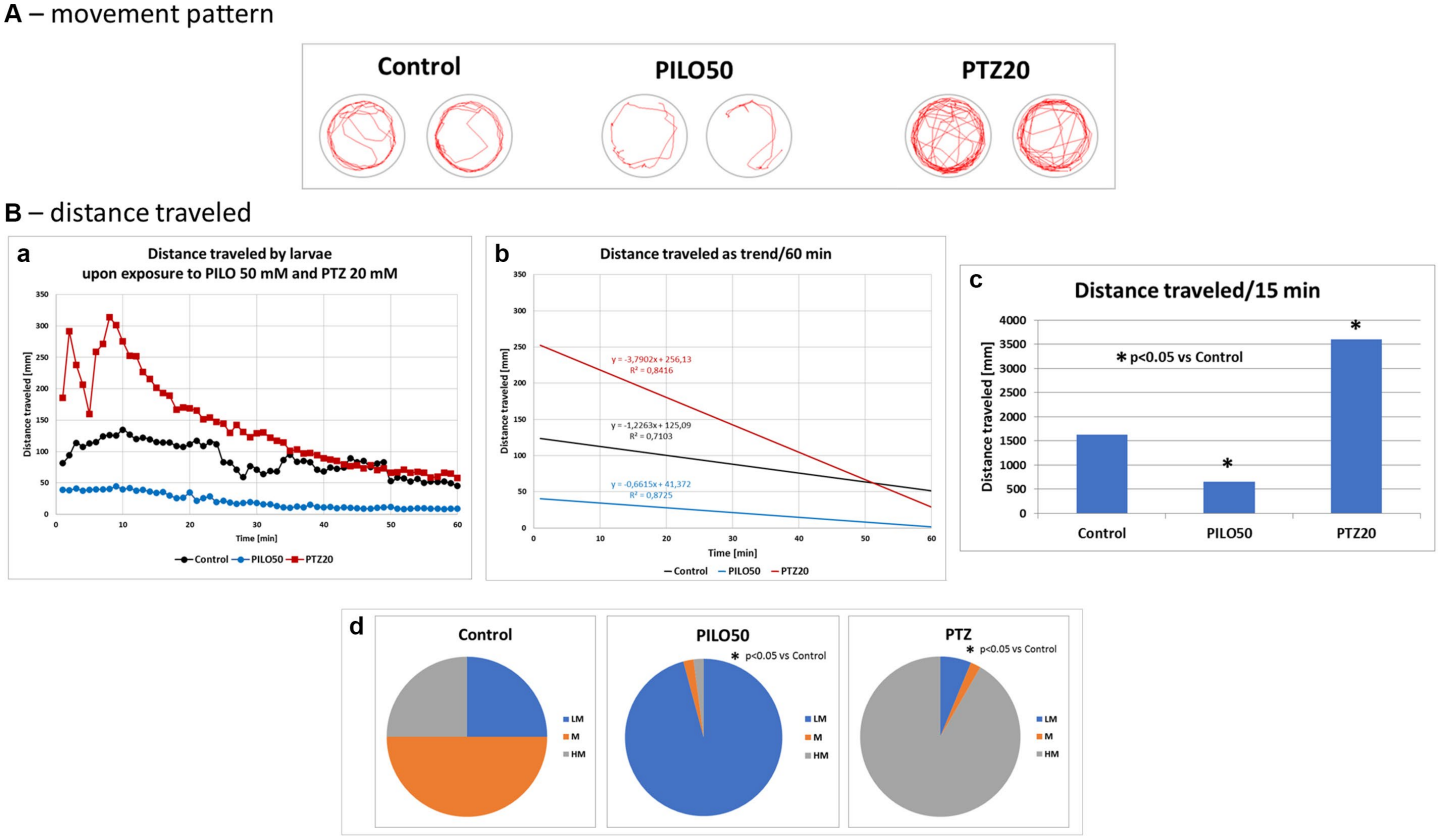
Comparison of locomotor activity of zebrafish larvae exposed to pilocarpine (PILO) or pentylenetetrazole (PTZ). Larvae were incubated in medium containing PILO (50 mM) or PTZ (20 mM) for 5 min, and, afterwards, locomotor activity assay was conducted for 60 min at 1 min intervals: **(A)** representative track visualizations which show the manner and intensity of zebrafish larvae movement in the well area (red line); the analysis was conducted at 9–10 min trial, the time of highest activity of larvae exposed to PTZ. **(B,a)** Each point represents median value of distance traveled at 1 min intervals; **(b)** locomotion was presented as a linear trend—the equation *y* = *mx* + c and squared value *R*^2^ are given in the graph; **(c)** total distance traveled in 60 min (data are presented as median values)—statistical analysis was performed using nonparametric ANOVA Kruskal–Wallis test, followed by Dunn’s Multiple Comparisons test (significance level was set at *p* < 0.05); **(d)** the pie charts show the fractions of larvae in quartiles: LM—larvae with low total mobility, M—larvae with moderate total mobility, HM—larvae with high total mobility; statistical analysis was performed using chi-square (*χ*^2^) statistics (significance level was set at *p* < 0.05, number of subjects *N* = 48/group).

Acute exposure of zebrafish larvae to PILO (50 mM) or PTZ (20 mM) induced a decrease and increase of locomotor activity as compared to control, respectively. When the movement pattern of the larvae was tracked by track visualization analysis it was found that, unlike PTZ, PILO-exposed larvae performed few whirlpool-like movements (Figure 7A). PILO, unlike PTZ, reduced distance traveled by larvae already from the beginning of exposure (Figure 7B,a). In contrast, PTZ induces a rapid increase in activity in the first 10–20 min followed by its decline to control levels (Figure 7B,a). The slope of the trend line of mobility affected by PILO is almost parallel to the control, while the trend for larvae exposed to PTZ has a different course (Figure 7B,b). The effects produced by both drugs were statistically significant compared to control group (Figure 7B,c). The further quartile analysis of larvae mobility revealed that acute exposition to PILO and PTZ reduces and increases the proportion of subjects with higher movement behavior, respectively (Figure 7B,d).

### 3.8 Histological consequences of exposure of zebrafish larvae to pilocarpine

**Figure 8:**
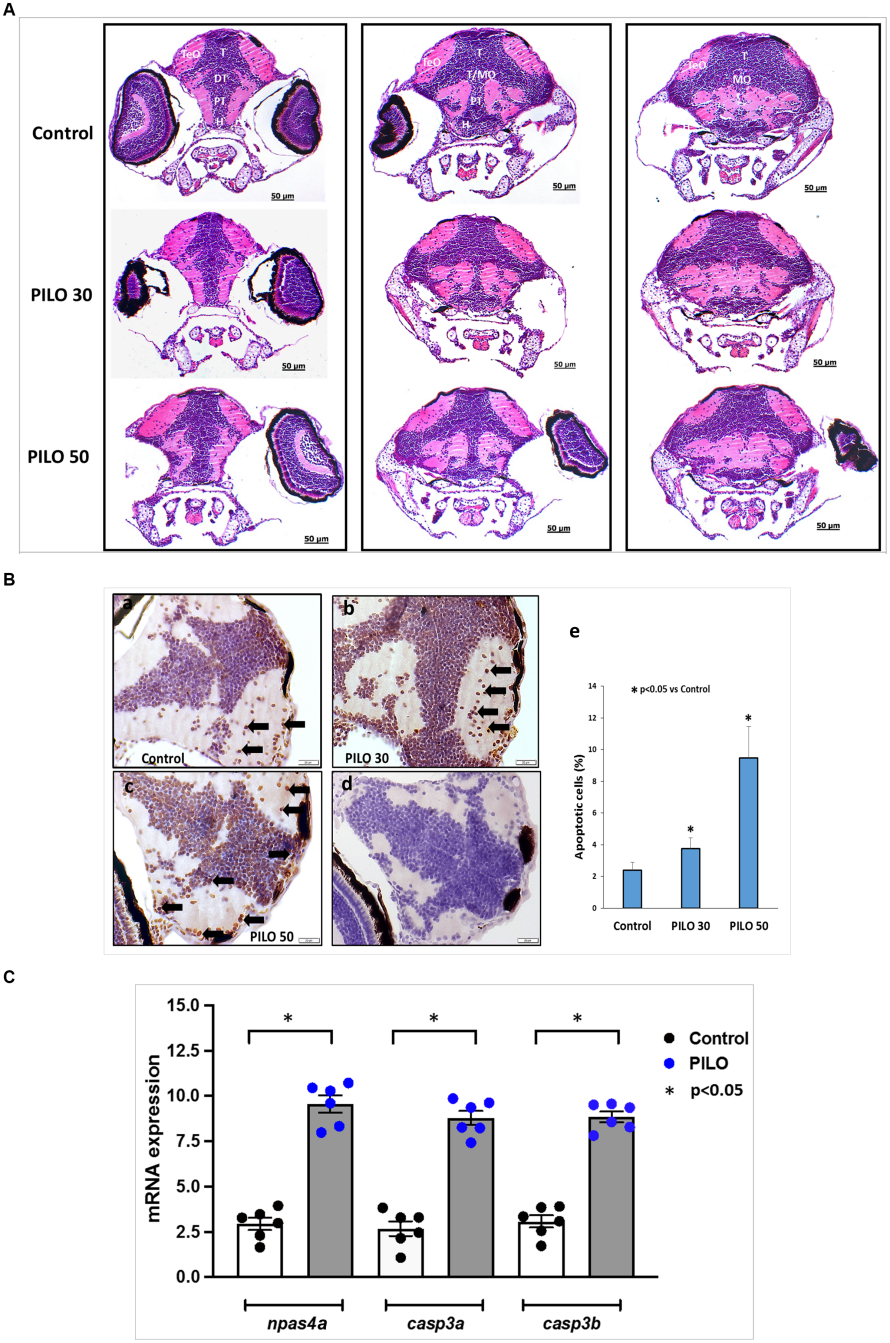
Histological changes in the brain of zebrafish larvae exposed to pilocarpine (PILO). **(A)** Brain morphology under hematoxylin and eosin (H&E) staining—larvae were incubated in medium containing PILO (30 or 50 mM) for 2 h. Next, larvae were thoroughly washed out × 5 times and incubated in fresh medium for subsequent 22 h. Afterwards, larvae were cooled down and fixed in 10% buffered formalin for 24 h at room temperature and processed for further analysis. DT, dorsal thalamus; H, hypothalamus; MO, medulla oblongata; PT, posterior tuberculum; T, midbrain tegmentum; TeO, tectum opticum; T/MO, midbrain tegmentum/medulla oblongata boundary. **(B)** Apoptosis evaluated by means of TUNEL staining. Larvae were incubated in medium containing PILO (30 or 50 mM) for 2 h. Next, larvae were thoroughly washed out × 5 times and incubated in fresh medium for subsequent 22 h. Afterwards, larvae were cooled down and fixed in 10% buffered formalin for 24 h at room temperature and processed for further analysis: **(a–c)** TUNEL staining, arrows indicate representative TUNEL-positive cells marked as dark brown; **(d)** a negative control was performed without active TdTenzyme; **(e)** the percentage of apoptotic cells over the total cell number per section per larva—the data are presented as the means ± standard deviations (SDs) (*N* = 5/group)—a one-way ANOVA test, followed by Dunnett’s multiple comparison analysis, were used for statistical evaluation (the significance was set at *p* < 0.05). **(C)** mRNA expression of *npsa4a* (marker of neuronal activity), *casp3a* and *casp3b* (markers of apoptosis). Larvae were incubated in medium containing PILO (50 mM) for 2 h. Next, larvae were thoroughly washed out × 5 times and incubated in fresh medium for subsequent 22 h. Next, samples were collected (*n* = 6 per group, *N* = 25 larvae per sample).

Morphological studies on the brain of zebrafish larvae were conducted 22 h after exposure to 30 mM and 50 mM PILO. H&E staining did not reveal the presence of necrotic lesions and areas of neurodegeneration (Figure 8A). In the TUNEL study, a significant increase of the number of apoptotic cells in the brain was found in larvae exposed to 30 and 50 mM PILO (Figure 8Ba–c,e).

Analysis of expression of genes on mRNA level conducted 22 h post-exposure to 50 mM PILO showed a statistically significant elevation of expression of the marker of neuronal activity, *npas4a*, and two markers of apoptosis *casp3a* and *casp3b* (Figure 8C).

## 4 Discussion

Our results indicate that PILO induces seizures in zebrafish larvae, as evidenced by recurrent, paroxysmal abnormal brain electrical activity recorded as a change in LFP. The isolated attacks evolve, from short duration and low amplitude events, to prolonged, frequently recurring, high amplitude electrographic alternations which resemble *status epilepticus*. This activity is completely inhibited by DZP, a drug used to prevent or terminate seizures. Prolonged recurring seizure events brought about by PILO initiate the process of epileptogenesis, with increased apoptosis occurring in the brain of zebrafish larvae. This is followed by alterations in the brain that result in unprovoked, repetitive seizures and distinct behavioral changes. Thus, the results obtained allow us to conclude that PILO induces seizures in zebrafish larvae, which can progress to *status epilepticus* and initiate the process of epileptogenesis, leading to the generation of morphological and/or functional reorganization of the brain and enabling the occurrence of recurring spontaneous seizures.

The sequence of events recorded in zebrafish larvae exposed to PILO resembles the behavioral, electroencephalographic and morphological changes that appear after intracerebral or systemic administration of PILO and other cholinomimetics in rodents (Turski et al., 1983a, 1983b, 1984). The process of epileptogenesis and unprovoked seizures also occurs in rodents (Cavalheiro et al., 1991, 1994). Noteworthy, these phenomena were described in adult individuals, while PILO was found to be less effective in young animals (Cavalheiro et al., 1987; Rose Priel et al., 1996; Curia et al., 2008).

The demonstration of the occurrence of epileptogenesis-like changes at such an early stage of brain development and maturation in zebrafish larvae provides a unique opportunity to track the phenomena leading to early childhood epilepsy. An additional advantage of the phenomenon of epileptogenesis in zebrafish larvae is the short time that elapses from the insults to the onset of unprovoked/spontaneous seizures, which, in our experiments, developed within 24 h, as confirmed by LFP recordings and *npas4a* overexpression. In rodent studies, the period of epileptogenesis lasts from 5 to 45 days (Cavalheiro et al., 1994; Curia et al., 2008). It should also be emphasized the lack of toxicity of PILO at a concentration of 50 mM and its 100% effectiveness in inducing spontaneous convulsions. In the case of PILO-induced convulsions in rodents, the mortality rate during *status epilepticus* and in the first few days reaches 27–85%, and spontaneous convulsions do not occur in all animals (Turski et al., 1989; Curia et al., 2008). More favorable results are achieved in the lithium-PILO model of epilepsy (Ahmed Juvale and Che Has, 2020), but the presence of lithium in high concentrations in the cells can interfere with the pathophysiological processes studied, complicating their unambiguous interpretation. Other procedures involve administration of PILO several times in lower doses and/or cessation of the *status epilepticus* by administration of either DZP or a cocktail of seizure-interrupting and relaxant drugs (Curia et al., 2008; Ahmed Juvale and Che Has, 2020).

Our study also demonstrates that, in contrary to PTZ-induced convulsions in larval zebrafish which behaviorally display as increased locomotor activity (Afrikanova et al., 2013; Moradi-Afrapoli et al., 2017; Gawel et al., 2020), PILO-induced seizures manifest in the form of decreased locomotion measured as distance traveled. Our observations, however, do not corroborate with the findings of Szep et al. who showed that in their experimental setup, both PTZ and PILO increased animal locomotor activity. What is, however, important, Szep et al. applied a different methodology, that is, they utilized a much longer accommodation period of 30 min between exposure to the substance and the start of the analysis (Szep et al., 2023). In our experiments, the accommodation period lasted 5 min. It should also be noted that in our study, the most significant differences in motility occurred in the first period after PILO exposure, i.e., between the 5th and 25th minutes, a period that was not analyzed by Szept et al. It is also unclear which strain of fish was used by Szept et al., and the authors did not perform LFP recordings or molecular analysis (Szep et al., 2023). Other groups of researchers also reported that PILO brings about increase in bouts in zebrafish larvae, but, again, there are substantial differences in the methodology of behavior assessment performed by us and others. In the study by Vermoesen et al., the duration of observation lasted only 1 min and was preceded by 1 min of accommodation (Vermoesen et al., 2011). In addition, time moved was counted, but the distance traveled by the larva was not measured—as in our study. In the study by Lopes et al., the number of quadrants transitions (crossings) was scored, but the distance traveled was not measured. Moreover, the trial covered 18 min and began immediately after PILO addition to the medium (Lopes et al., 2016). In addition, in both publications, quantitative estimation was done by observer through visual inspection. In our study, we evaluated the behavior of larvae after exposure to PILO in detail and in several ways. We also analyzed the distance traveled in 1 min intervals and the total distance traveled during the 60 min of the trial. In addition, we evaluated the pattern of larvae behavior through quartile analysis. All analyses consistently indicated that exposure of larvae to PILO causes a concentration-dependent reduction of locomotion measured as distance traveled.

Similarly to our results, kainic acid, which is known to induce seizures progressing to *status epilepticus* in rodents (Lévesque and Avoli, 2013), causes chronic epileptic state in larval zebrafish with decreased locomotor activity (Heylen et al., 2021). Collectively, our results and data from previous studies demonstrate that, convulsions in larval zebrafish manifest differentially from PTZ-induced seizures. Hence, it is fundamental to standardize and unify the way of assessing PILO-induced seizure activity in zebrafish larvae if this model is to be used in future studies on epilepsy, as the effects of antiseizure medications and the processes of epileptogenesis, and the evaluation will be performed based on behavioral changes.

To further analyze the pattern of larvae behavior during exposure to PILO, we quantified not only the distance traveled, but also the time spent in motion (moving time) and the speed of movement (velocity), which was calculated by dividing the distance traveled by the time spent moving during the recording period, i.e., 1 min. As expected, the moving time was reduced, but to our surprise, the speed of movement was not reduced, but statistically higher than in the control larvae. This was also the case with larvae investigated 22 h after the end of exposure to PILO. These observations reveal that PILO did not disrupt the larvae’s motor apparatus, but rather point to a change in brain activity. This type of analysis has never been reported before and therefore we propose to use all three parameters as an algorithm for behavioral assessment of PILO-induced seizures. In this context, movement velocity appears to be particularly useful and informative because it is highly sensitive in terms of statistical significance judgment. Our proposal needs to be confirmed in further studies and, most importantly, needs to be verified by assessing what effect antiseizure medications exert on the behavioral parameters we have indicated in larvae exposed to PILO.

The zebrafish models of seizures and epilepsy are currently widely applied for screening purposes (Gawel et al., 2020; Knap et al., 2023). For example, *scn1lab*^−/−^ mutants (a model of Dravet syndrome) have served as a screening platform and have pinpointed clemizole as a potential drug candidate for Dravet syndrome patients (Baraban et al., 2013), and more recently also for *STXBP1* mutations carriers (Moog and Baraban, 2022). The translational value of PTZ-induced seizure model in larval zebrafish is seen for cannabinoids (Samarut et al., 2019; Thornton et al., 2020) and currently used antiseizure medications (Afrikanova et al., 2013). Zebrafish seizure models also have provided data for anticonvulsant activity of antiparkinsonian drug lisuride (Sourbron et al., 2019), an active ingredient of ginger, i.e., 6-gingerol (Gawel et al., 2021) or the resveratrol analog, pterostilbene (Nieoczym et al., 2019). Furthermore, the antiepileptogenic properties of fenfluramine, potent serotonin-enhancing molecule, was shown by Tiraboschi et al. in *scn1lab*^−/−^ mutants (Tiraboschi et al., 2020). Therefore, we propose that our model may serve as a new screening platform for identification of new molecules with anticonvulsant activity.

The PILO-induced model of seizures in rodents is already employed for investigation of epileptogenesis processes. In this regard the unique advantage of zebrafish is ease, compare to rodents, of genetic manipulation and the relatively short period of time of obtaining mutants. Zebrafish genome has been sequenced, and 70% of human genes has at least one orthologue in zebrafish genome (Howe et al., 2013). A rich toolbox allows investigating changes at molecular and cellular levels with relative ease and pace. Indeed, the epileptogenic process has been investigated, e.g., in *scn1lab*^−/−^ (Tiraboschi et al., 2020) or *tsc2*^−/−^ mutants (model of tuberous sclerosis complex) (Kedra et al., 2020). Considering our model, the whole process of obtaining seizing larvae for examination, is very short (2 h), easy (incubation), and cheap. Since most of the cases of epilepsy occurs in two extremities of life—in children and the elderly—our model especially allows investigating epileptogenic processes in very early developmental periods (childhood epilepsy). This is not achievable using PILO in rodents.

### 4.1 Limitations

As potential study limitations, it is worth noting that the study did not evaluate content of PILO in larvae 22 h post exposition to the drug. Therefore, it cannot be ruled out that the electroencephalographic and behavioral changes in larvae recorded 22 h after PILO exposure and subsequent washing out period are the result of PILO residues in the larvae’s body. However, it seems unlikely, as PILO is water soluble and does not accumulate noticeably even after its prolonged administration in humans (Seal et al., 2022; Kannarr et al., 2023).

Our study did not examine whether unprovoked convulsions persist for an extended period of time after exposure to PILO. Since zebrafish larvae demonstrated a high capacity for organ regeneration, an interesting exploratory aspect may be the assessment of repair capacity of the brain to insult induced by convulsive activity.

## 5 Conclusion

In this study, we show that PILO induces seizures in zebrafish larvae, as evidenced by recurrent, paroxysmal abnormal brain electrical activity recorded as a change in LFP. A convulsive state induced by PILO initiates the process of epileptogenesis, with increased apoptosis occurring in the brain of zebrafish larvae. This is followed by alterations in the brain that result in unprovoked, repetitive seizures and behavioral changes. Therefore, we propose the PILO-induced seizure and epilepsy model as a new platform for screening purposes in the search for epileptogenesis modifying drugs, as well as in basic research on epileptogenesis phenomena.

## Data availability statement

The raw data supporting the conclusions of this article will be made available by the authors, without undue reservation.

## Ethics statement

Ethical approval was not required for the study involving animals in accordance with the local legislation and institutional requirements because the larvae up to 5 days post-fertilization were used only.

## Author contributions

KG: Conceptualization, Data curation, Formal analysis, Funding acquisition, Investigation, Methodology, Project administration, Resources, Software, Supervision, Validation, Visualization, Writing – original draft, Writing – review & editing. MH-S: Formal analysis, Investigation, Methodology, Resources, Writing – review & editing. MM-G: Formal analysis, Investigation, Methodology, Writing – review & editing. AG: Formal analysis, Investigation, Methodology, Writing – review & editing. AS: Formal analysis, Methodology, Writing – review & editing. NK: Investigation, Writing – review & editing. WE: Investigation, Writing – review & editing. CE: Resources, Writing – review & editing. PK: Resources, Writing – review & editing. WAT: Conceptualization, Data curation, Funding acquisition, Methodology, Resources, Writing – original draft, Writing – review & editing.
